# Effects of different factors on the friction and wear mechanical properties of titanium alloy materials with cortical bones at near service conditions

**DOI:** 10.1371/journal.pone.0290346

**Published:** 2023-10-19

**Authors:** Xingdong Sun, Jianfei Zhou, Ke Xu, Wandi Wu, Liangyuan Xu, Rui Jiang, Liangfei Fang

**Affiliations:** School of Engineering, Anhui Agricultural University, Hefei, China; University of Vigo, SPAIN

## Abstract

The artificial joint is one of the most effective methods to treat joint injuries. The service performance of artificial joints is gradually weakened because of the wear of artificial joints in actual service. In order to obtain the potential failure mechanism of the artificial joint in actual service, the study was carried out with the multiple factors that affect the service performance of the artificial joint. The experimental study was carried out on the change rule of mechanical behavior of the contact interface between the artificial joint of titanium alloy and cortical bone. The multi-factor is compression load, contact load, friction velocity, and lubrication environment, respectively. The results indicate that the friction coefficient, wear mass, and wear coefficient of Ti-6A1-4V titanium alloy decreased with the increasing of the compression load. The friction rate and the friction coefficient of Ti-6A1-4V titanium alloy decreased with the increasing of the contact load. The wear mass and friction coefficient of Ti-6A1-4V titanium alloy increased with the increasing of contact load. The lubrication effect is better with the increasing of lubricant concentration. Based on the observation of the SEM, the wear type influenced by compression load and friction rate is mainly abrasive wear and oxidation wear. The wear type influenced by contact load is mainly abrasive wear and adhesive wear. The wear type influenced by lubricants is mainly oxidation wear. When wear mass and wear coefficient are used as the criteria for evaluating friction and wear, the order of influential factors to friction and wear of Ti-6Al-4V titanium alloy plate is friction rate, compression load, contact load, and lubricant concentration. This research can provide a theoretical reference for the optimal manufacture of the artificial joint of titanium alloy and optimal rules of safe service conditions.

## 1. Introduction

Recently, artificial hip joint replacement has increased by 174% in China [1]. Artificial joint replacement is one of the most effective treatments for joint injuries [2]. The friction pair between the artificial joint implanting into the human body and the nature joint withstands friction cycles of one to three million every year [3]. Harmful ions produced in the wear process of the artificial metal joint can cause aseptic loosening, neurological diseases and other problems [4–8]. Therefore, friction and wear of artificial joints are essential criteria for quality and service life [9]. Based on vital signs of friction and wear of artificial, domestic and foreign scholars have conducted extensive relevant research. Pengfei et al [10] found that the wear mass of Ti-6A1-4V titanium alloy is positive relevance to load and friction distance, and wear and friction in the fluid environment of the human body can be simulated by using different kinds of lubricants. Wang et al [11] found that the wear ratio and wear factor in dry friction of Ti-6A1-4V titanium alloy is higher than those in wet friction. Deng [12] found that the friction coefficient significantly decreased by the proper concentration of hydroxylated carbon nanotube coating material.

Currently, the friction and wear research of medical Ti-6A1-4V titanium alloy mainly focuses on the material itself. It is less that research friction and wear of contact interface between the artificial joint of implanting into the human body and cortical bone. The behavior of friction and wear in the service condition is an essential reason for the serving failure of the artificial joint.

Therefore, based on the actual service conditions of artificial joints, this study analyzed the effects of compression load, contact load, friction rate, and lubricant concentration on the friction and wear performance of the interface between artificial joints and cortical bone, and qualitatively analyzed the weight ratios of different influencing factors.

## 2. Materials and methods

### 2.1 Sample preparation

**Fig 1:**
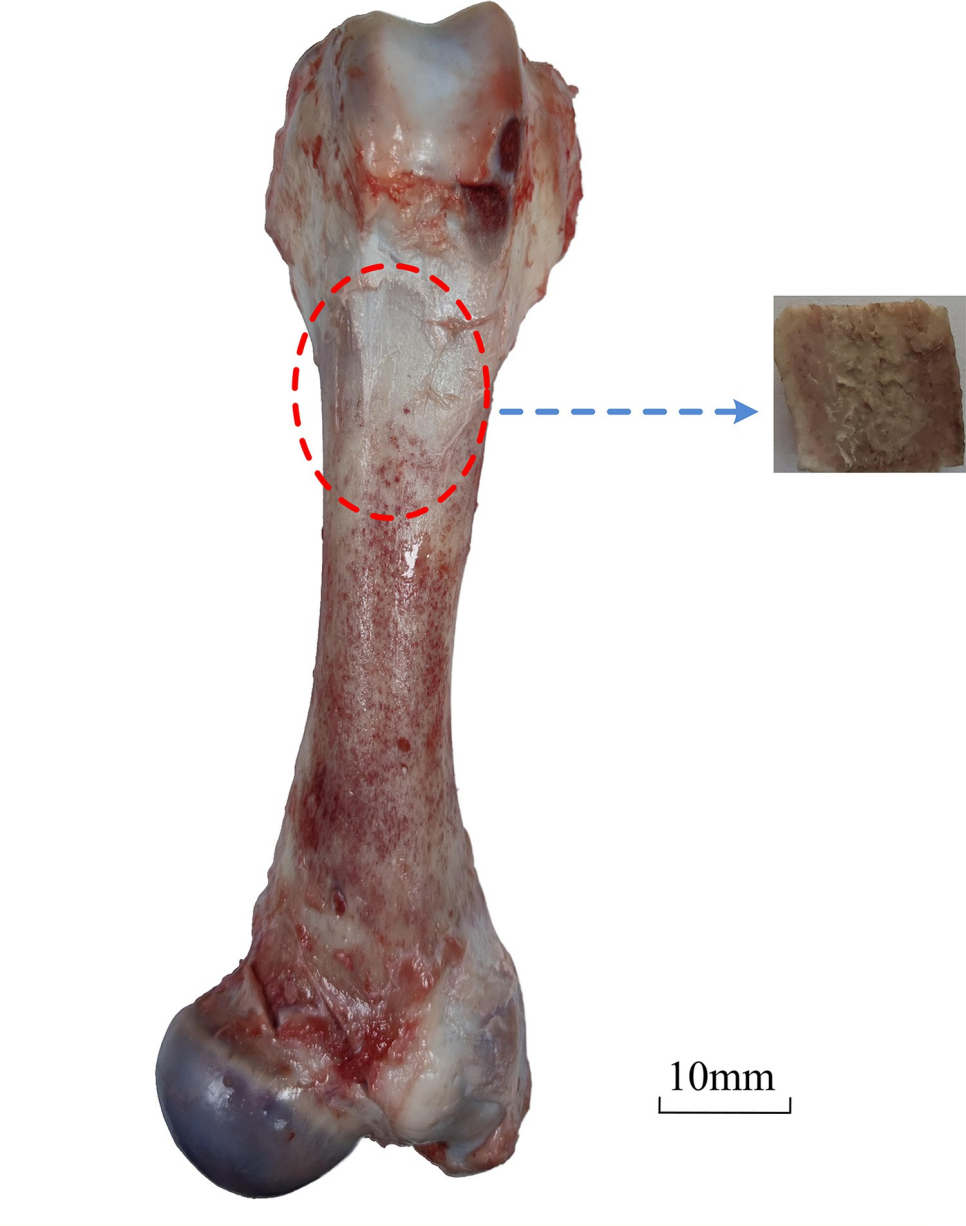
Sampling process of cortical bone sample.

The experimental goal simulated friction and wear of the femoral stem of the artificial hip joint to implant into the human body. The mechanical properties and structure of pig bone and human bone were similar [13]. The friction head’s size of the contact area and surface shape could affect frictional force [14]. The femur of the pig was used to replace the femur of the human body. The femur of pig was obtained from a 1-year-old white pig of Anhui Shunhua Food Company Limited. The friction head is the bone of the flat middle part of the pig femur. The size parameters of bone specimens and titanium alloy specimens are reported in Table 1. The position of the friction head was shown in Fig 1.

**Table 1:** Dimension parameters of the experimental specimens.

Sample name	Length (mm)	Width (mm)	Thickness (mm)
Specimen of Bone	12	12	5
Specimen of Titanium alloy test	270	40	5

**Fig 2:**
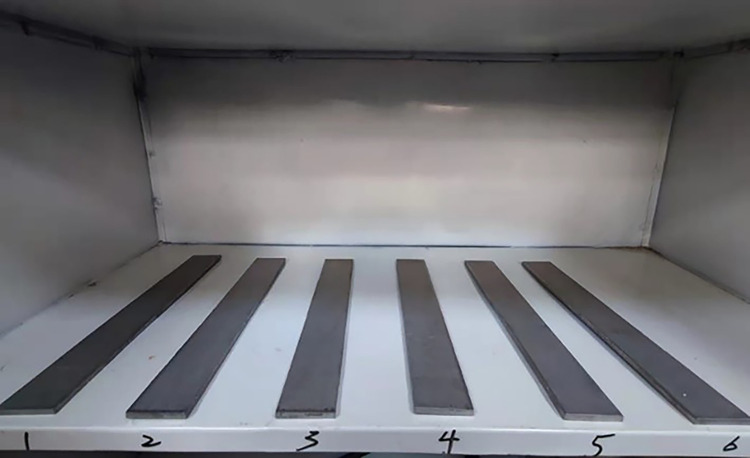
Picture of titanium alloy specimen.

The tested samples were medical Ti-6Al-4V titanium alloy (Baoji Yingtili Metal Materials Company Limited). The tested samples were shown in Fig 2 and fulfilled the standard of GB/T13810-2017. The Ti-6Al-4V titanium alloy plate was polished by the diamond grinding paste of WO. 5. Then, the surface of the Ti-6Al-4V titanium alloy plate was washed with an alcoholic cleaning solution.

### 2.2 Methods and test apparatus

**Fig 3:**
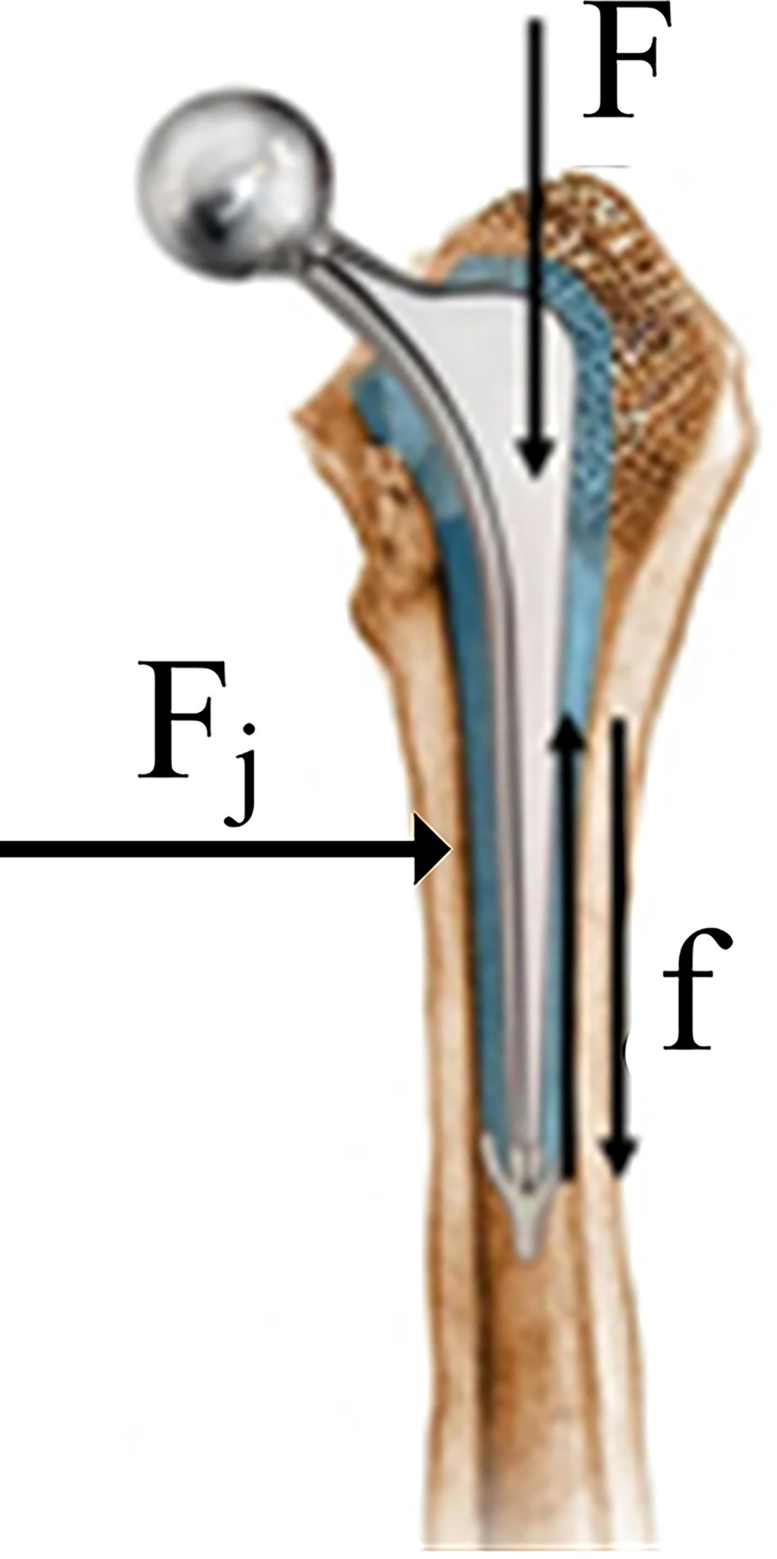
Internal force diagram of femoral stem of artificial hip joint. F: Compression load F_j_: Contact load f: Fiction force.

During actual service, the femoral stem of the artificial hip joint bore the compression load from body weight or activity, as shown in Fig 3.

**Fig 4:**
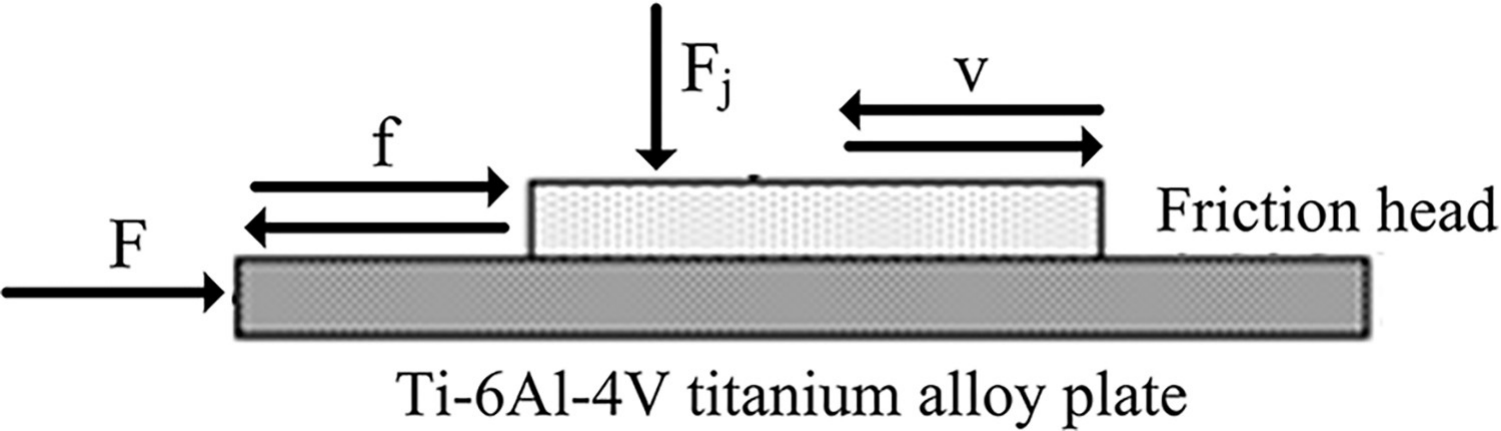
Schematic diagram of internal force on femoral stem of artificial hip joint. F: Compression load; F_j_: Contact load; f: Fiction force.

The contact zone between the femoral stem of the artificial hip joint and the wall of the bone cavity existed a frictional force in the vertical direction, because the contact zone existed contact load. As shown in Fig 4, the force acting of the stem of the artificial hip joint in the human body was simulated.

**Fig 5:**
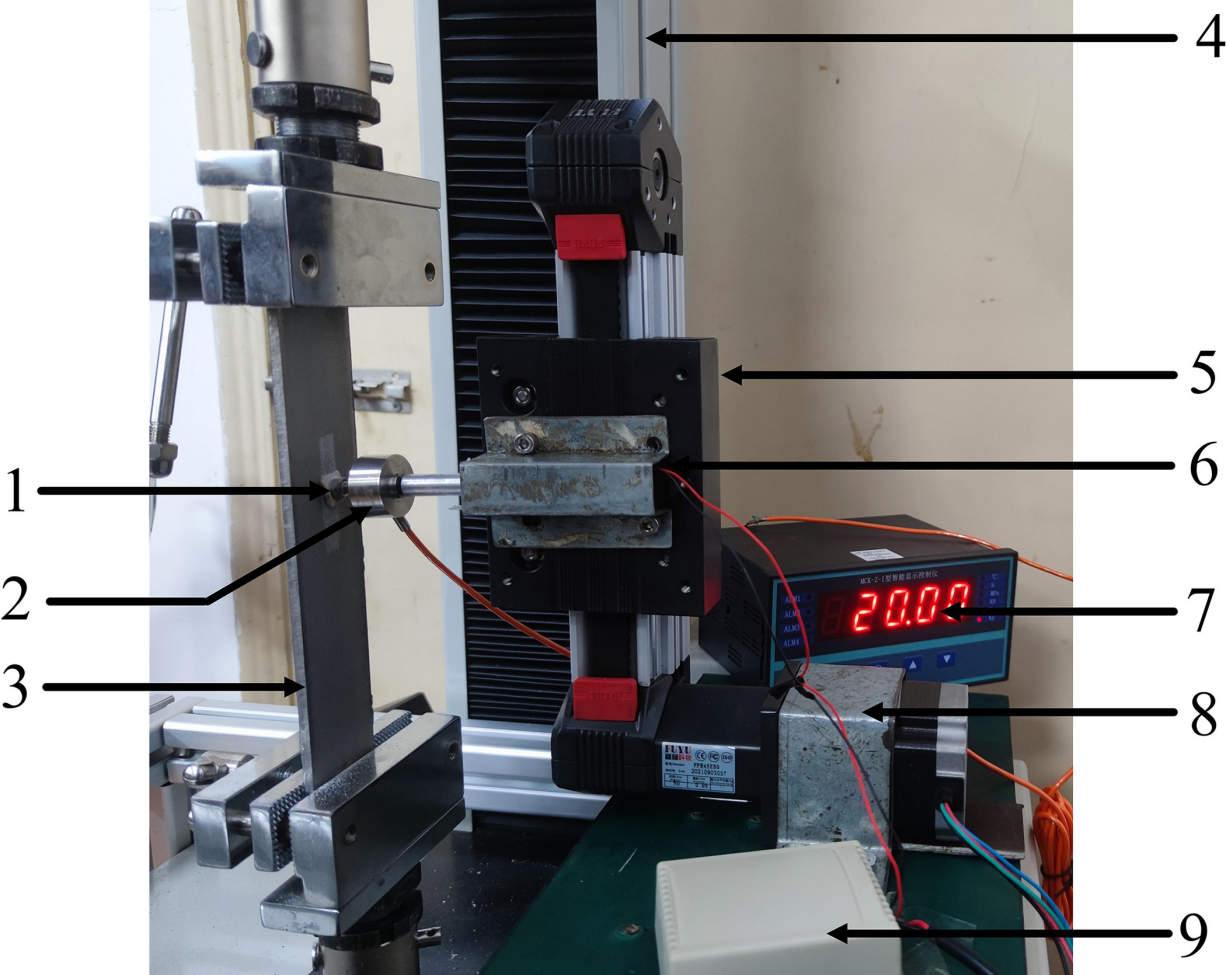
Friction and wear test device. 1: Friction head of bone 2: Pressure sensor 3: Ti-6Al-4V titanium alloy plate 4: Electronic testing machine 5: Sliding module 6: Linear actuator 7: Intelligent display 8: Drive motor for sliding module 9: Control box of linear actuator.

As shown in Fig 5, the pre-compression mechanical test system of friction and wear mainly consists of nine parts. Table 2 shown relative information of different modules of the test system.

**Table 2:** Relevant parameters and functions of the experimental module.

Name	Type	Range	Resolution ratio
Electronic testing machine	KY-10KNW (Shanghai Kaiyan Testing Instrument Co., Ltd)	0~10000 N	1/5000000
Sliding module	FPB45E50-L-B57 (Chengdu Fuyu Technology Co., Ltd)	0~600 N	1/40000

Tables 3–5 shows separately experiment information on compression load, contact load, and sliding friction rate. Each group of tests was repeated three times. The friction time for each group of tests was 25 minutes, and the displacement of the friction head was 25 mm.

**Table 3:** Experimental parameters of friction and wear under different compression loads.

Group No	Compression load (N)	Contact load (N)	Friction rate (mm/s)	Lubricant concentration (g/ml)
A1	200	20	30	Dry friction
A2	300	20	30	Dry friction
A3	400	20	30	Dry friction

**Table 4:** Experimental parameters of friction and wear under different contact loads.

Group No	Compression load (N)	Contact load (N)	Friction rate (mm/s)	Lubricant concentration (g/ml)
A1	200	10	30	Dry friction
A2	200	20	30	Dry friction
A3	200	30	30	Dry friction

**Table 5:** Experimental parameters of friction and wear under different friction rates.

Group No	Compression load (N)	Contact load (N)	Friction rate (mm/s)	Lubricant concentration (g/ml)
A1	200	20	30	Dry friction
A2	200	20	50	Dry friction
A3	200	20	70	Dry friction

The wear of the artificial joint resulted in an alteration of lubricants’ concentration and components and affected the wear performance of the artificial joint [15]. The different concentrations of lubricant were made by normal saline of 9% concentration and Bovine serum albumin [16]. Table 6 shows relative test parameters.

**Table 6:** Experimental parameters of friction and wear under different lubrication environments.

Group No	Compression load (N)	Contact load (N)	Friction rate (mm/s)	Lubricant concentration (g/ml)
A1	200	20	30	Dry friction
A2	200	20	30	0.1
A3	200	20	30	0.5

### 2.3 Analytical methods

The electro-mechanical testing machines offered a compressive load to the Ti-6Al-4V titanium alloy plate during the experiment. The electro-mechanical testing machines implemented displacement load keeping when the compressive load increased the predetermined value. During the displacement load keeping, the friction force between the Ti-6Al-4V titanium alloy plate and friction head led to the change of compressive load of the electro-mechanical testing machines. The contact load between the titanium alloy plate and the bone friction head is constant. The value of friction force was a difference between the initial displacement load value and the current load value of the electro-mechanical testing machines [17]. As shown in the Eq (1):

$$\mu =\frac{F_{r}‐F_{z}}{W}$$ (1)

Where *μ* is friction coefficient between Ti-6Al-4V titanium alloy plate and friction head; *F*_*r*_ is the load measured by the electronic testing machine in the experiment (N); *F*_*z*_ is the precompression load (N); *W* is contact load(N).

Before and after the experiment, the weight of Ti-6Al-4V titanium alloy was measured by the electronic balance (Shanghai Huachao Electric Appliance Company Limited). The mass loss converted into a wear factor was used to clearly reveal the wear resistance of Ti-6Al-4V titanium alloy in different experimental conditions [18]. As shown in Eqs (2) and (3):

$$△m=m_{1}‐m_{2}$$ (2)

$$K=\frac{△m}{\rho \cdot W\cdot S}$$ (3)

Where *m*_1_ and *m*_2_ are respectively the mass of Ti-6Al-4V titanium alloy plate before and after the experiment (g); △*m* is the weight loss of Ti-6Al-4V titanium alloy plate (g); *ρ* is the density of Ti-6Al-4V titanium alloy plate (g/cm^3^); *K* is wear coefficient (mm^3^/N·m); *W* is contact load(N); *S* is the sliding distance (m).

After the experiment, the surface topography of Ti-6Al-4V titanium alloy was observed by the scanning electron microscope (Hitachi, S-4800). It showed the wear mechanism, wear failure mode and wear resistance of Ti-6Al-4V titanium alloy. The friction coefficient, wear mass, and wear coefficient were the average value of the three experiments’ results.

## 3. Results and discussion

### 3.1 Effect of different test variables on friction and wear

**Fig 6:**
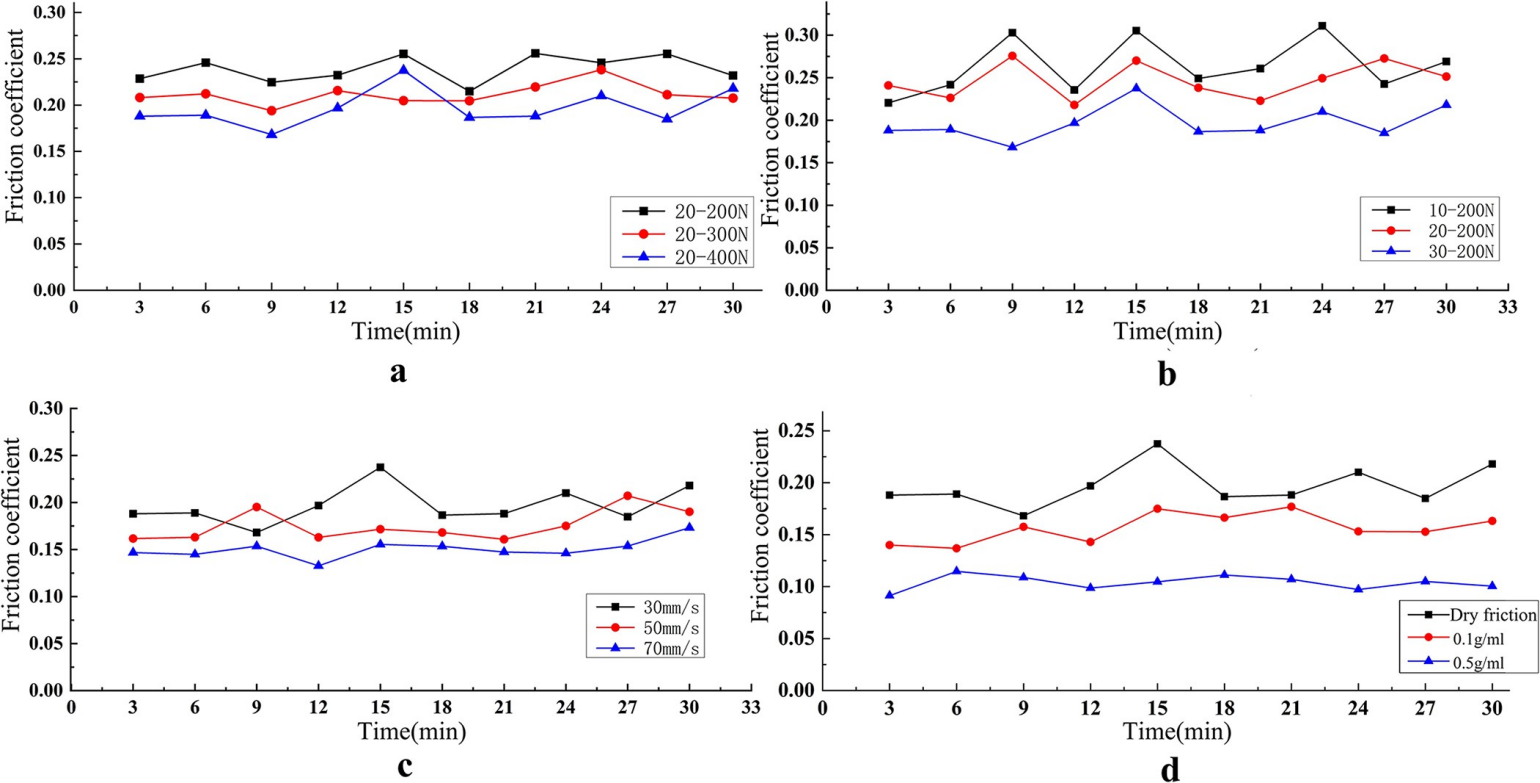
Effect of different experimental variables on average friction coefficient. (a) Different compression loads (b) Different contact loads (c) Different friction rates (d) Different lubrication environments.

As shown in Fig 6, the average friction coefficient curve of different compression load, contact load, friction rate, and lubricant condition is shown separately. The friction coefficient decreases with an increasing of compression load, contact load, friction rate, and lubricant concentration by comparing with the change of each curve.

The average wear mass and average wear coefficient on different experimental variations was shown in the Table 7. Wear mass decreased with the increasing of the compression load, and friction rate increased with the increasing of the contact load. The wear coefficient decreased with the increasing of the compression load, contact load, and friction rate. Compared with dry friction, lubricant can significantly reduce the value of the wear mass and wear coefficient.

**Table 7:** Average wear mass and average wear factor under different experimental variables.

Group No	Average wear mass (g)	Average wear factor (mm^3^/N·m)	Group No	Average wear mass (g)	Average wear factor (mm^3^/N·m)
A1	2.1025	0.009344	A2	1.8377	0.008167
A3	1.1612	0.005161	B1	1.8710	0.016631
B2	2.3599	0.006992	C1	1.7552	0.007801
C2	1.0682	0.004748	D1	1.2936	0.00575
D2	0.9454	0.004202			

Under compressive load, dislocations, and tensile twinning occur in the Ti-6Al-4V titanium alloy plate, resulting in higher surface hardness of the Ti-6Al-400V titanium alloy plate [19]. The Ti-6Al-4V titanium alloy plate mainly undergoes bending and compression loads under the compression load. Due to the compression load being much greater than the bending load, the Ti-6Al-4V titanium alloy plate mainly undergoes bending deformation. Therefore, the friction coefficient, wear mass, and wear coefficient all decrease with increasing compressive load. The friction coefficient (*μ*) is negatively related to the contact load (*W*) based on the adhesive wear theory and the friction contact theory of Hertz [20]. The pressing depth of the friction head into the Ti-6Al-4V titanium alloy plate and the furrow degree of the Ti-6Al-4V titanium alloy plate increased with increasing of the contact load. Therefore, wear mass is significantly increase with the increasing of contact load, too. Because the meshing degree of the micro convex body in the contact area between the bone friction head and Ti-6Al-4V titanium alloy plate decreases with the increase of friction rate, [21] the friction coefficient under low friction rate is bigger than that under high friction rate. The higher friction rate is better for timely removing friction debris. The heat generated by a high friction rate can generate the oxide thin film on the surface of the Ti-6Al-4V titanium alloy plate, and the oxide thin film can increase the wear resistance of the Ti-6Al-4V titanium alloy plate. Lubricants can generate a lubricating film on the surface of the friction pair. Because the high concentration lubricants are better on covering the friction pair surface, it is benefit to decrease the wear properties of the Ti-6Al-4V titanium alloy plate.

### 3.2 Effect of different variables of surface morphology

**Fig 7:**
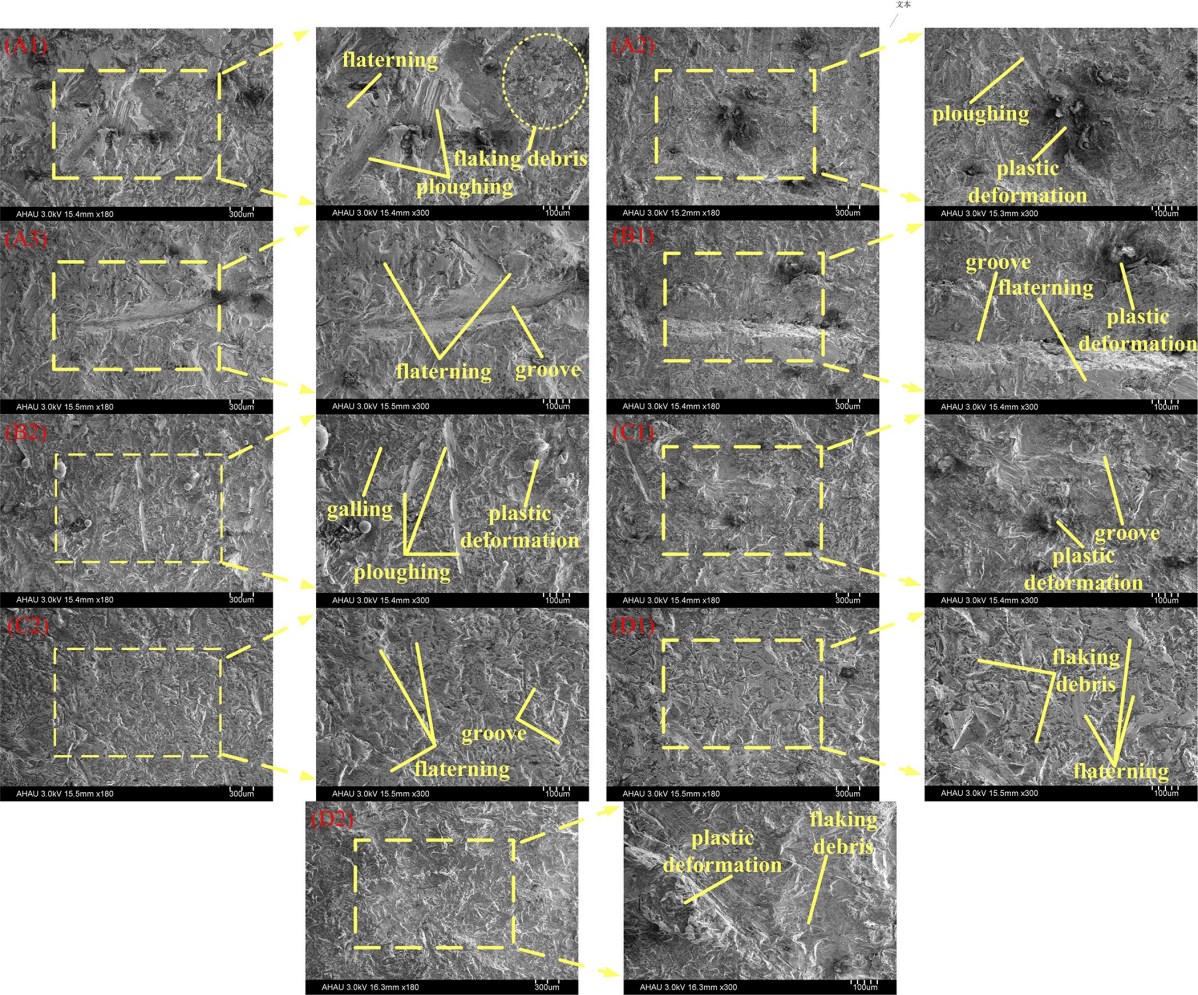
Effect of different experimental variables on surface morphology of Ti-6Al-4V titanium alloy plate. (A1) is the sample photo with test group No. A1 (A2) is the sample photo with test group No. A2 (A3) is the sample photo with test group No. A3 (B1) is the sample photo with test group No. B1 (B2) is the sample photo with test group No. B2 (C1) is the sample photo with test group No. C1 (C2) is the sample photo with test group No. C2 (D1) is the sample photo with test group No. D1 (D2) is the sample photo with test group No. D2.

As shown in Fig 7, the surface shape of the Ti-6Al-4V titanium alloy plate is affected by different experimental variations. The surface wear of the Ti-6Al-4V titanium alloy plate is gradually reduced with the increasing of compression load. The change of compression load mainly affects abrasive wear and oxidative wear. The decreased number of spalling pits verifies that the wear mass and wear coefficient decreased with the increasing of compressive load (A1, A2, A3). The furrow effect on the surface of the Ti-6Al-4V titanium alloy plate is gradually noticeable with the increase of contact load. The adhesive wear and large particle of wear debris have occurred with the increasing of the contact load. The wear types are mainly abrasive wear and adhesive wear with the increase of contact load (A1, B1, B2). Because high friction rates easily lead to an increased surface temperature of Ti-6Al-4V titanium alloy plate and produce a dense oxide thin film, the smoothness of the surface of Ti-6Al-4V titanium alloy plate increases with the increasing of the friction rate. The friction rate mainly affects the wear type of abrasive wear and oxidation wear (A1, C1, C2). The furrow marks on the surface of the Ti-6Al-4V titanium alloy plate on dry friction are typical abrasive wear and symbolize high wear consumption. After adding lubricants, the surface wear mechanism of Ti-6Al-4V titanium alloy plate mainly is oxidative wear and accompanies a small amount of abrasive wear (A1, D1, D2).

### 3.3 Qualitative analysis of different influencing factors

**Fig 8:**
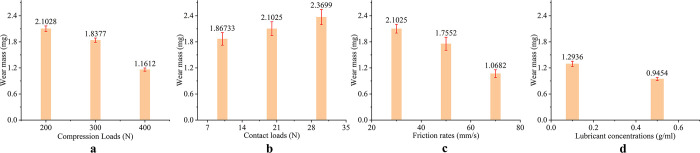
Influence of different factors on wear mass. (a) Different compression loads (b) Different contact loads (c) Different friction rates (d) Different lubrication environments.

**Fig 9:**
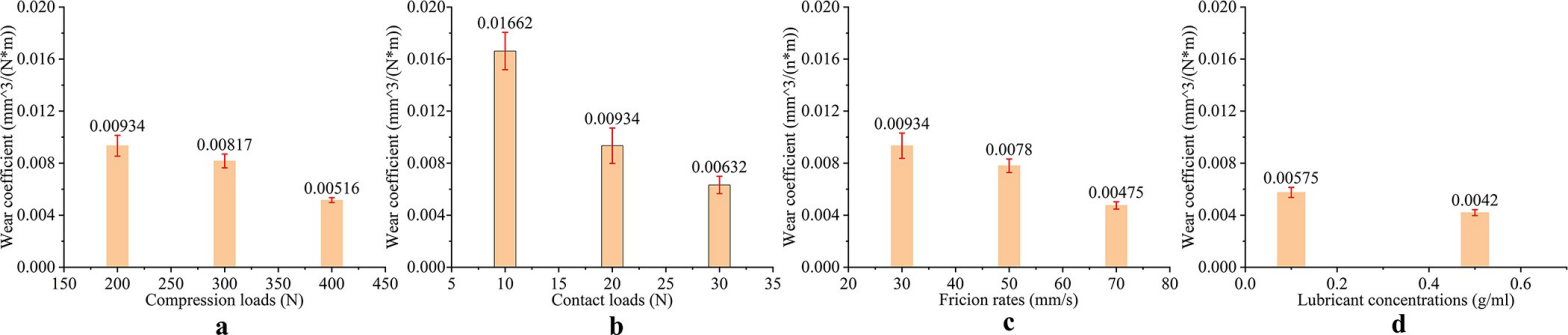
Effect of different factors on wear coefficient. (a) Different compression loads (b) Different contact loads (c) Different friction rates (d) Different lubrication environments.

As shown in Fig 8, the wear mass is affected by different influential factors. The influence of compression load and friction rate to wear mass is more significant than contact load and lubricant concentration to wear mass. When the compression load increased from 200 N to 400 N, the wear mass decreased by 44.8%. When the friction rate increased from 30 mm/s to 70 mm/s, the wear mass decreased by 49.2%. As shown in Fig 9, the wear coefficient is affected by different influence factors. The influence of contact load and friction rate to wear mass is more significant than compression load and lubricant concentration to wear mass. When the contact load increased from 10 N to 20 N, the wear coefficient decreased by 43.8%. When the friction rate increased from 30 mm/s to 70 mm/s, the wear factor decreased by 49.1%. Under compression load, the Ti-6Al-4V titanium alloy plate will undergo dislocation and tensile twinning, resulting in higher surface hardness of the Ti-6Al-4V titanium alloy plate. This can effectively reduce the contact area between the surface of the Ti-6Al-4V titanium alloy plate and the friction head, as well as surface cracks caused by wear, thereby reducing the average friction coefficient. According to Archard Eqs (4) and (5):

$$\frac{dV}{dS}=k_{m}{\left(1+\alpha f^{2}\right)}^{1/2}\beta \frac{W}{\sigma_{s}}$$ (4)

$$\frac{M}{\rho }=\frac{kFL}{H}$$ (5)

Where *V* is the wear volume (m^3^), *S* is the friction distance (m), *W* is the contact load between the friction pairs (N), *k*_*m*_ and *β* are respectively related to the fixed coefficient and the surface properties of the material, *α* is a constant, *F* is the coefficient of friction (N), *δ*_*s*_ is the compressive yield limit of the material (Pa), *M* wear amoun (kg), *ρ* is the density (kg/m^3^), *k* is a constant, *F* is the wear load (N), *L* is the friction distance (N), *H* is the Vickers hardness.

So, the wear volume (*V*) is directly proportional to the friction coefficient (*f*), and the wear amount (*W*) is inversely proportional to the hardness (*H*) of the material. When the Ti-6Al-4V titanium alloy plate is subjected to compression load, its hardness increases and the friction coefficient decreases, resulting in a decrease in wear amount and wear volume with the increasing of the compression load. The wear volume (*V*) is proportional to the contact load (*F*), and as shown in Table 7, the ratio of contact load to wear mass is greater than 1. Therefore, as the contact load increases, the wear factor decreases. According to the comprehensive analysis of the influence of different influential factors on wear quality and wear factor, the order of influential factors to friction and wear of Ti-6Al-4V titanium alloy plate is friction rate, compression load, contact load, and lubricant concentration.

## 4. Conclusions

According to the complexity and necessity of friction and wear mechanism on artificial joint materialism, the experimental study was carried out on the friction and wear mechanism of the contact interface between typical artificial joint materialism of Ti-6A1-4V titanium alloy and cortical bone under different influence factors. The conclusions are as follows:

(1) When the compression load is 200~400 N, the average friction coefficient and wear mass decrease with the increasing of the compression load. The wear type influenced by compression load is mainly abrasive wear and oxidation wear.

(2) When the contact load is 10~30 N, the average friction coefficient decreases with the increasing of the contact load, and the average wear mass increases with the increasing of the contact load. The wear type influenced by contact load is mainly abrasive wear and adhesive wear.

(3) When the friction rate is 30~70 mm/s, the average friction coefficient and average wear mass decrease with the increasing of the friction rate. The wear type influenced by friction rate is mainly abrasive wear and oxidation wear.

(4) After adding bovine serum albumin solution lubricants, friction coefficient and wear mass significantly decrease. The effect of decreasing wear in the 0.5 g/ml serum protein solution is better than in the 0.1 g/ml serum protein solution. The wear type of influence by lubricants is mainly oxidation wear.

(5) When wear mass and wear coefficient are used as the criteria for evaluating friction and wear, the order of influential factors to friction and wear of Ti-6Al-4V titanium alloy plate is friction rate, compression load, contact load, and lubricant concentration.
